# Aniracetam does not improve working memory in neurologically healthy pigeons

**DOI:** 10.1371/journal.pone.0215612

**Published:** 2019-04-19

**Authors:** Hannah Phillips, Arlene McDowell, Birgitte S. Mielby, Ian G. Tucker, Michael Colombo

**Affiliations:** 1 Department of Psychology, University of Otago, Dunedin, Otago, New Zealand; 2 School of Pharmacy, University of Otago, Dunedin, Otago, New Zealand; University of Modena and Reggio Emilia, ITALY

## Abstract

Understanding the effects of cognitive enhancing drugs is an important area of research. Much of the research, however, has focused on restoring memory following some sort of disruption to the brain, such as damage or injections of scopolamine. Aniracetam is a positive AMPA-receptor modulator that has shown promise for improving memory under conditions when the brain has been damaged, but its effectiveness in improving memory in neurologically healthy subjects is unclear. The aim of the present study was to examine the effects of aniracetam (100mg/kg and 200 mg/kg) on short-term memory in “neurologically healthy” pigeons. Pigeons were administered aniracetam via either intramuscular injection or orally, either 30 or 60 minutes prior to testing on a delayed matching-to-sample task. Aniracetam had no effect on the pigeons’ memory performance, nor did it affect response latency. These findings add to the growing evidence that, while effective at improving memory function in models of impaired memory, aniracetam has no effect in improving memory in healthy organisms.

## Introduction

Cognitive enhancing drugs (nootropics) are drugs that can improve memory and cognition, either by increasing attention or enhancing the mechanism by which memory occurs [1]. Cognitive enhancers are of particular interest as a way to counter disruptions to memory, such as that caused by aging or traumatic brain injury. A less explored avenue of research, however, is the effect of cognitive enhancers on healthy brains. Caffeine is a commonly used cognitive enhancer that improves wakefulness and energy [2] due to its psychostimulant properties. Other psychostimulants, such as cocaine or methylphenidate (Ritalin), have also demonstrated a facilitative effect on memory-based tasks [3, 4], possibly due to increasing a subject’s ability to focus.

Cognitive enhancers can also target the α-amino-3-hydroxy-5-methyl-4-isoxazolepropionic acid (AMPA) receptor that plays an important role in the formation of long-term potentiation [5], a purported molecular mechanism by which memory occurs. AMPAkines are a group of drugs that positively modulate the AMPA receptor, slowing receptor gating so that the receptor stays open for longer [6], leading to facilitated long-term potentiation. In behavioural studies, use of AMPAkines increased the performance of rats on spatial memory tasks [7] and the performance of monkeys on a delayed matching-to-sample (DMS) tasks [8]. One AMPAkine with considerable evidence for its cognitive enhancing effects is piracetam. Day-old chicks injected with 10 mg/kg piracetam demonstrated an enhanced ability to learn a passive avoidance task over chicks given saline [9]. In another study with rats, 250 mg/kg of acute piracetam administration did not improve DMS performance, but when 250 mg/kg were given chronically over 11 days matching accuracy did improve [10]. Piracetam also restores memory in both rats and mice following damage to the brain by either injections of scopolamine and induction of abnormal levels of CO_2_ in the blood [10, 11].

Aniracetam is another AMPAkine created by modifying the piracetam molecule by replacing the acetamide moiety with a 4-methoxybenzoyl group [12]. Aniracetam has few side effects (e.g. anxiety, insomnia, diarrhoea), a wide therapeutic window and a short half-life, making it very safe [13, 14], and has demonstrated a restorative effect in animal models of impaired memory. Aniracetam improves memory to healthy levels following disruption via scopolamine injection [13, 15, 16], sleep deprivation [17], surgical brain damage [18, 19], or as part of normal aging [14, 18, 20, 21]. Aniracetam also has a protective effect when administered prior to disruption. When administered before testing, aniracetam protects against memory loss caused by electrical brain stimulation [11, 22], haloperidol administration [23], and CO_2_-induced learning impairment [11].

Prior evidence suggests that aniracetam may also improve healthy memory. In a DMS task, aniracetam improved matching accuracy in monkeys during the longer delay lengths in a dose-dependent manner, such that 25 mg/kg aniracetam produced the greatest improvement, and 50 mg/kg produced a far more modest improvement when compared with saline [16]. Additionally, aniracetam improved healthy rats’ performance on active avoidance tasks [13], and on radial arm maze tasks [24].

Only one study has previously examined the effects of aniracetam in pigeons [16]. Pigeons were injected with 12.5, 25 or 50 mg/kg aniracetam 50 minutes before testing on a DMS task. While matching accuracy did increase slightly, the improvement was not statistically significant [16]. There are two main reasons why aniracetam may not have had a significant effect on performance. First, the doses chosen (12.5, 25, and 50 mg/kg) may have been too low to have an effect in pigeons. Although in monkeys these dosages did have an effect [16], the optimal dosage in rodents reported in other studies is more in the 100–300 mg/kg range [15, 23], and it is possible that as in rodents, higher dosages may be required in pigeons for the effects of aniracetam to be noticed. Second, given that aniracetam has a short half-life of about 30 min [25] and that the pigeons were tested 50 minutes after administration, it is possible that the small doses of aniracetam may have already been metabolised by the time testing occurred. Thus, a primary motivation behind the current study was to address both of these issues. We tested a higher dose of aniracetam (100 mg/kg and 200 mg/kg) in pigeons prior to performance on the DMS task, and the drug was administered either 30 or 60 minutes prior to the DMS task to account for the short half-life. Additionally, we also tested both oral and intramuscular routes of administration.

## Materials and method

### Subjects

The subjects were 14 unsexed adult pigeons (*Columba livia*). They were housed individually and fed a diet of mixed grains at an amount designed to maintain them at 80–85% of their free-feeding weight. The birds were kept on a light/dark cycle of 12 hours starting at 7am. The subjects were kept and treated in accordance with the University of Otago Code of Ethical Conduct for the Manipulation of Animals, and the University of Otago Animal Ethics Committee approved the experiment.

### Apparatus and stimuli

**Fig 1:**
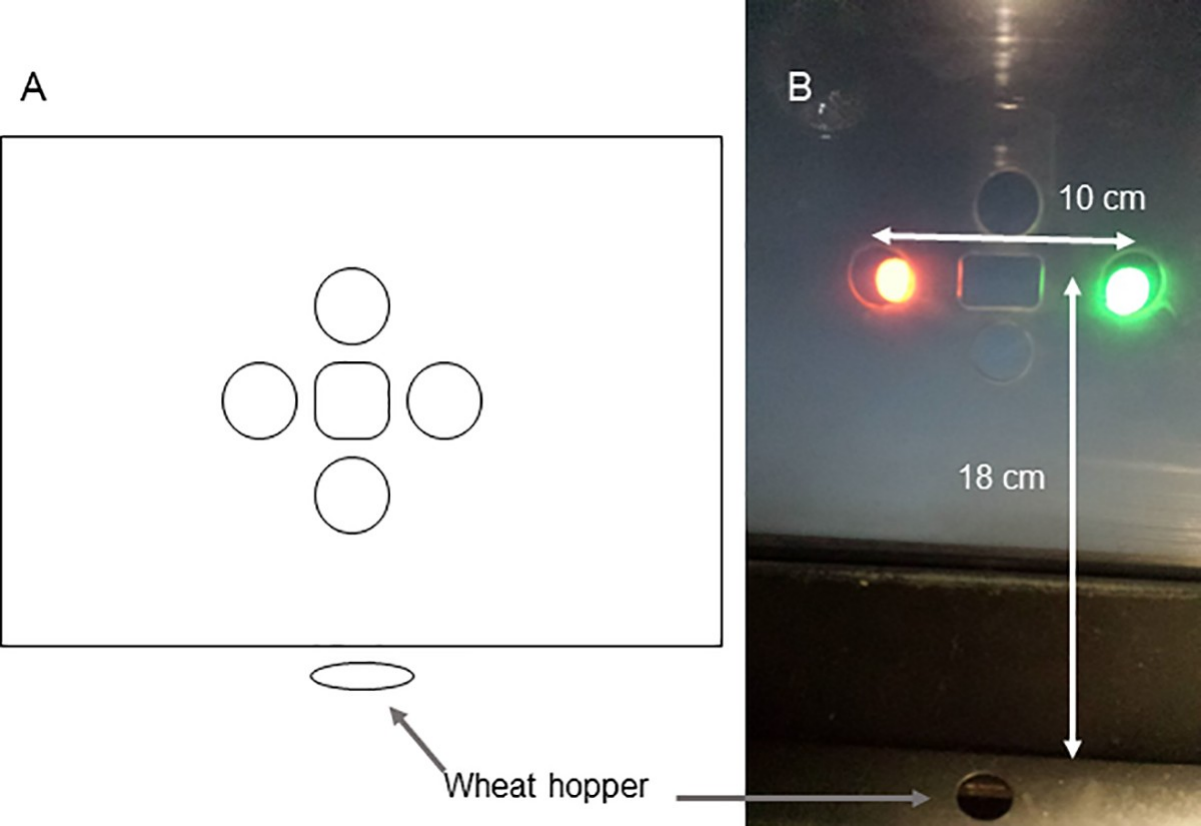
**Illustration (A) and photograph (B) of the apparatus.** Only the left, center, and right apertures were used in the experiment. Shown is the presentation of the red and green comparison stimuli. Food (wheat) was delivered via a hole below the display and at floor level.

Training and testing occurred in 45 cm x 45 cm x 36 cm operant chambers equipped with touchscreen monitors, upon which stimuli were displayed. In front of the touchscreen monitors was a Plexiglas panel with four circular holes (2.5 cm in diameter) surrounding a single rectangular hole (3.5 cm x 2 cm; Fig 1A). The two side circular holes were each 10.5 cm from the edge of the screen to their centre, and the top vertical holes was 16.5 cm from the top of the chamber, and the bottom circular hole was 18 cm from the floor. The distance between the edge of the central rectangular hole and the inner edge of the side holes was 2 cm and 0.5 cm from the inner edge of the vertical holes (top and bottom).

The stimuli were either a circular red or green coloured disc, 1.7 cm in diameter (Fig 1B). In the current study, only the two side holes and the central hole were used to present stimuli. Wheat served as a reward and was delivered by a hopper positioned below the screen. The distance from the edge of the central hole to the hopper was 20 cm. The hopper was illuminated while it was raised.

### Training phase

The pigeons were first autoshaped to peck a red or green disc displayed in the central hole to obtain wheat. Once trained to peck at the stimuli for food, they were trained on the delayed matching-to-sample (DMS) task. The DMS procedure was as follows. A sample stimulus (either a red or green disc) was presented in the central hole. Once the bird had pecked the sample stimulus three times the stimulus turned off and a variable delay began. At the end of the delay, both red and green stimuli were presented in the side holes. A correct response was defined as the pigeon selecting the comparison stimulus that matched the just-seen sample stimulus. Doing so resulted in a reward of 1.4 sec access to wheat, followed by a 15 sec intertrial interval (ITI). Selection of the incorrect comparison stimulus resulted in no reward and a 15 sec timeout from the task, followed by a 15 sec ITI.

A session consisted of 64 trials. With red and green discs as the stimuli and the sample stimulus presented in the central hole and the comparison stimuli presented in the side holes, there were four possible sample-comparison configurations: RRG, GRR, RGG and GGR. Red and green served equally often as the sample stimulus. The position of the red and green comparison stimuli was balanced, such that there were 16 trials dedicated to each of the four sample-comparison configurations.

DMS training began with only one delay, initially with 0 sec delay inserted between the offset of the sample and the onset of the comparison stimulus. The criterion for increasing the delay to .5 sec and then 1 sec was a performance level of 13/16 (81.2%) or more correct responses for each of the four sample-comparison configurations across two consecutive days. Once the performance criterion was achieved with the 1 sec delay the birds were moved onto DMS training with variable delays starting with 0, 1, 2, and 4 sec (Delay set 1). All sessions again consisted of 64 trials with eight trials dedicated to each delay for each of the red and green sample stimuli (e.g., red 0 sec, red 1 sec, red 2 sec, red 4 sec, green 0 sec, green 1 sec, green 2 sec, green 4 sec). In order to move on to the next delay set, performance at 0, 1, 2 and 4 sec needed to be at least 7/8, 6/8, 6/8 and 5/8 correct responses, respectively, for each trial type, for two consecutive days.

The pigeons were then cycled through up to four additional delay sets (Delay set 2: 0, 2, 4, 8 sec; Delay set 3: 0, 3, 6, 9 sec; Delay set 4: 0, 4, 8, 16 sec; and Delay set 5: 0, 5, 10, 20 sec). There was a rolling criterion for moving from one delay set onto the next. Performance for each of the delays was summed over three consecutive days, giving a total of 24 trials for each delay. The criterion for advancing to the next delay set was 21/24 (87.5%), 18/24 (75%), and 15/24 (62.5%) correct responses for the first three delays for two consecutive blocks of three days. There was no performance criterion for the longest delay. If a bird did not reach criterion within 30 days, the current delay set was selected as the delay set to be used for the bird’s drug trials.

### Intramuscular administration of aniracetam

**Fig 2:**
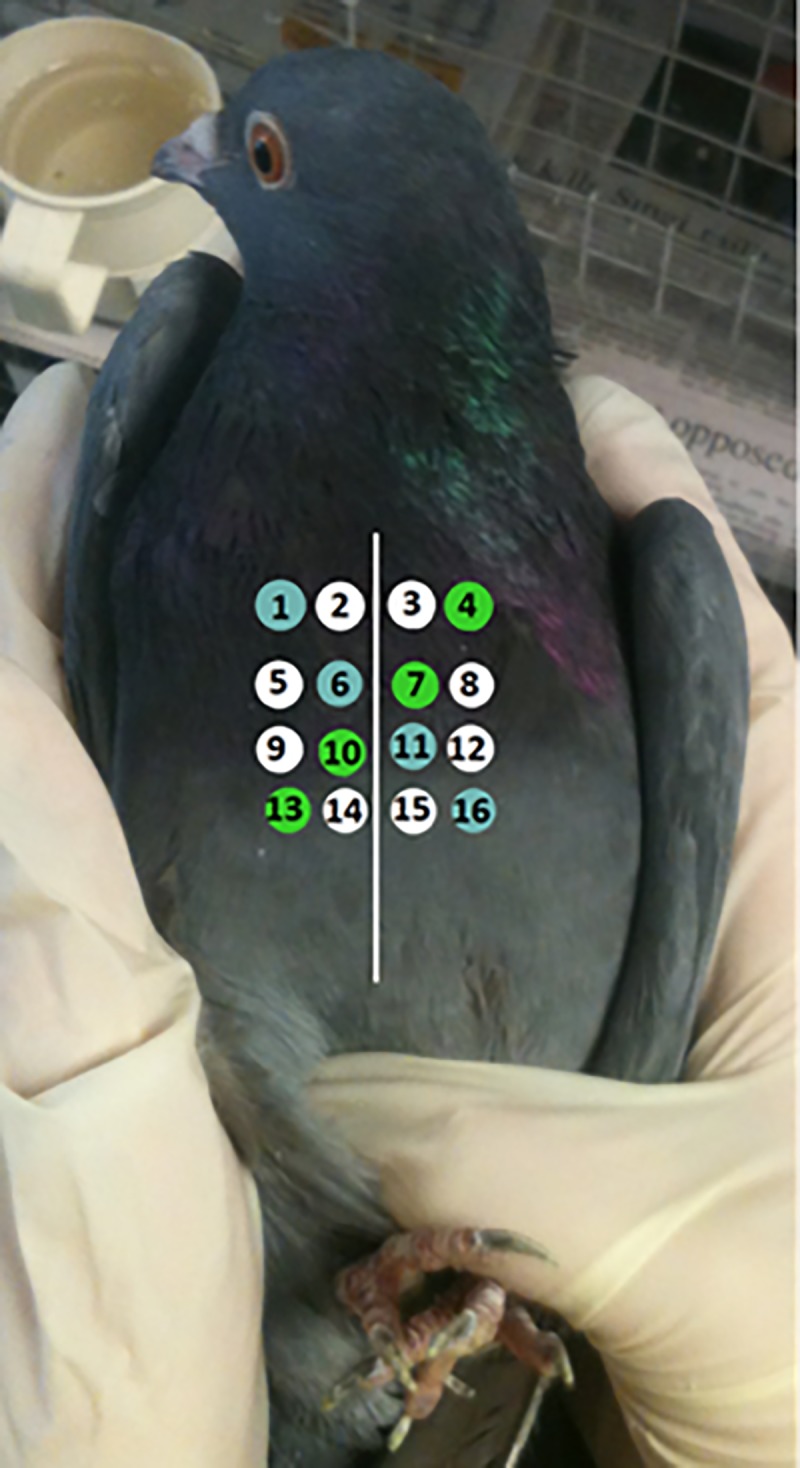
Position of the 16 intramuscular injection sites on the breast of the pigeon. Green and blue coloured sites indicate positioning of the 100 mg/kg and 200 mg/kg doses. White injection sites were reserved for saline injections.

Aniracetam was prepared as a suspension with Tween 80 and saline in a ratio of 1:9. The vial containing the suspension was placed into a warm water bath between 104–120°F for 45 minutes, then placed in an ultrasonic bath for 15 minutes to disperse any aniracetam agglomerates to allow for easier passage through a 23 gauge needle when injected. Prior to each injection, the vial was again placed in the ultrasonic bath for five minutes. There were 16 proposed injection sites for each bird, eight on each side of the keel. These sites are shown in Fig 2.

The order of the injection was such that the saline, 100 mg/kg, and 200 mg/kg injections were spaced across the injection sites in a counterbalanced arrangement (Fig 2). For each bird, the 100 mg/kg and 200 mg/kg injection sites were each arranged along a diagonal line, reaching from either top left to bottom right (blue injection sites in Fig 2) or top right to bottom left (green injection sites in Fig 2). Whether the 100 mg/kg dose or the 200 mg/kg dose fell on the blue or green injection sites was counterbalanced between birds. The saline injections were arranged around the remaining eight injection sites such that the number of injections on each side of the keel was equal.

The pigeons were divided into two groups; those who were injected 30 minutes prior to testing (referred to as Inject-30, n = 6), and those who were injected 60 minutes prior to testing (referred to as Inject-60, n = 8) and were placed onto a 24 day drug administration schedule in which they received an injection every second day (Table 1). Each bird received an equal amount of saline, 100 mg/kg, and 200 mg/kg injections across this 24-day schedule. On the days where no injection was administered, the birds were still tested on the DMS task as a control condition. The birds were not tested during the weekend.

**Table 1:** Timetable of drug administration for each bird.

Days	Day 1	Day 2	Day 3	Day 4	Day 5	Day 6/7
1–6	100 mg/kg	NI	200 mg/kg	NI	Saline	REST
7–12	200 mg/kg	NI	Saline	NI	100 mg/kg	REST
13–18	Saline	NI	200 mg/kg	NI	100 mg/kg	REST
19–24	Saline	NI	100 mg/kg	NI	200 mg/kg	REST

On the first five days of each week the animal is tested on the DMS task, whereas on the weekend the animal is not tested on the DMS task and given a “REST”. “NI” indicates a day in which the animal is tested on the DMS task but no injection is given.

### Oral administration of aniracetam

The injection study was conducted first followed by the oral administration study. The birds were divided into two groups, a 30-minute group (referred to as Oral-30) and a 60-minute group (referred to as Oral-60). All the birds in the oral administration study had participated in the injection study and served in the oral study in a balanced fashion. Specifically, three of the Inject-30 birds went on to serve in the Oral-30 study, and the other three went on to serve in the Oral-60 study. Similarly, two of the Inject-60 birds went on to serve in the Oral-30 study, and two other Inject-60 birds went on to serve in the Oral-60 study.

Holes were drilled into individual kernels of corn such that the corn was open at both ends and hollow in the middle. Aniracetam (70 mg–corresponding to a 200 mg/kg dose in a bird of average weight) was mixed with 0.1 mL distilled water until it formed a paste, which was then loaded into a single corn kernel. Birds in the Oral-30 and Oral-60 groups were fed a single aniracetam-filled corn kernel either 30 or 60 minutes prior to testing, respectively. Birds were fed aniracetam-filled corn kernels every second day, and plain corn kernels (without aniracetam) every other day for a total of 12 days. Three birds stopped eating the aniracetam-filled corn after a few days. For these birds, the beak was held gently open, the kernel placed in their mouth, and their beak closed until the kernel was swallowed. Because of time constraints, oral administration was only tested using the 200 mg/kg dosage.

### Statistical analyses for inject and oral data

The Inject-30 and Inject-60 DMS retention and latency data were both subjected to a repeated-measures two-way ANOVA with drug concentration (4: no injection, saline, 100 mg/kg aniracetam, 200 mg/kg aniracetam) and delay (4: delays 1–4) as factors, with Greenhouse-Geisser correction for repeated measures. The Oral-30 and Oral-60 DMS retention and latency data were both subjected to a repeated-measures two-way ANOVA with drug (2: no drug, 200 mg/kg aniracetam) and delay (4: delays 1–4) as factors with Greenhouse-Geisser correction for repeated measures.

### Pharmacokinetic study

Ten birds participated in the 100 mg/kg pharmacokinetic group (three from the Inject-30 group, one from the Inject-60 group, and six novel birds) and a different ten birds participated in the 200 mg/kg pharmacokinetic group (two from the Inject-30 group, four from the Inject-60 group, and four novel birds). Aniracetam (100 or 200 mg/kg) was administered orally as a powder in a pre-drilled corn kernel as described above. Blood samples (0.2 mL) were withdrawn from a brachial wing vein at either -5, 5, 10, 15, 30, 45, 60, or 90 min after dosing and collected into heparinized Eppendorf tubes. Blood was drawn from the birds in both the 100 mg/kg and 200 mg/kg groups anywhere from 1–4 of the possible 8 time-points, quasirandomly determined, and in such a way that all time-points were sampled. On average, birds in the 100 mg/kg group had blood withdrawn 2.88 times compared to 2.78 times for the bids in the 200 mg/kg group. The body weight of birds was between 235 g and 428 g. The birds were fasted for 24 h and had water available ad libitum.

Blood samples were separated by centrifugation for 5 min at 11337 g. The plasma was aspirated and stored at -80°C until analysis. Protein extraction of the plasma samples was based on Ogiso et al. [25] and was carried out by adding 60 μL acetonitrile (99.9%) purchased from Merck (Darmstadt, Germany) containing 100 ng/mL N-methyl-2-pyrrolidione (99.5%), purchased from Sigma-Aldrich (St. Louis, MO, USA) to 30 μL of plasma. Samples were mixed in a vortex and separated by centrifugation for 10 min at 9660 g.

LC-MS/MS analysis was performed using an Agilent Technologies 6430 Triple Quadrupole LC/MS System with Jet Stream ionization based on the method of Cai and Wang [26]. The separation of aniracetam and N-anisoyl-GABA was achieved using a Synergi 4 μm Fusion RP80Å Phenomenex column (150 x 4.6 mm). The isocratic mobile phase consisted of an organic phase (0.1% formic acid in acetonitrile) and an aqueous phase (0.1% formic acid in HPLC water) in ratio 30:70 v/v. The injection volume was 10 μL. The conditions of the LC-MS/MS were capillary voltage 4000 V, source temperature 300°C and the desolvation gas was nitrogen with a flow rate of 11 L/min. The optimized collision energy was 12 V for aniracetam and N-anisoyl-GABA, and 24 V for N-methyl-2-pyrrolidione (the internal standard). Aniracetam, N-anisoyl-GABA and N-methyl-2-pyrrolidione were quantified by multiple reaction monitoring in positive ion mode using the precursor ion → product ion combinations of *m/z* 220.1 → 135.1, *m/z* 238.1 → 135.1 and *m/z* 100.1 → 58.1, respectively. There was a linear relationship between response and concentration of analytes over the range 2.5–500 ng/mL. Linear regression analysis of standard curves produced correlation coefficients > 0.996. The precision of the method for aniracetam at three QC levels was < 14% and the accuracy < 14.3% (*n* = 3). The precision of the method for N-anisoly-GABA was < 17% and the accuracy < 11% (*n* = 3). The lower limit of detection (LLOD) and lower limit of quantification (LLOQ) for aniracetam in plasma were 1.79 ng/mL and 5.41 ng/mL, respectively. The LLOD and LLOQ for N-anisoyl-GABA were 1.07 ng/mL and 3.23 ng/mL, respectively.

The plasma concentration-time data after oral administration of aniracetam were analysed by noncompartmental pharmacokinetic methods performed using Phoenix 64 (WinNonlin, Certara LP). The area under the concentration-time curve (AUC) up to the last sampling point was calculated using the trapezoidal method.

## Results

### Inject-30

**Fig 3:**
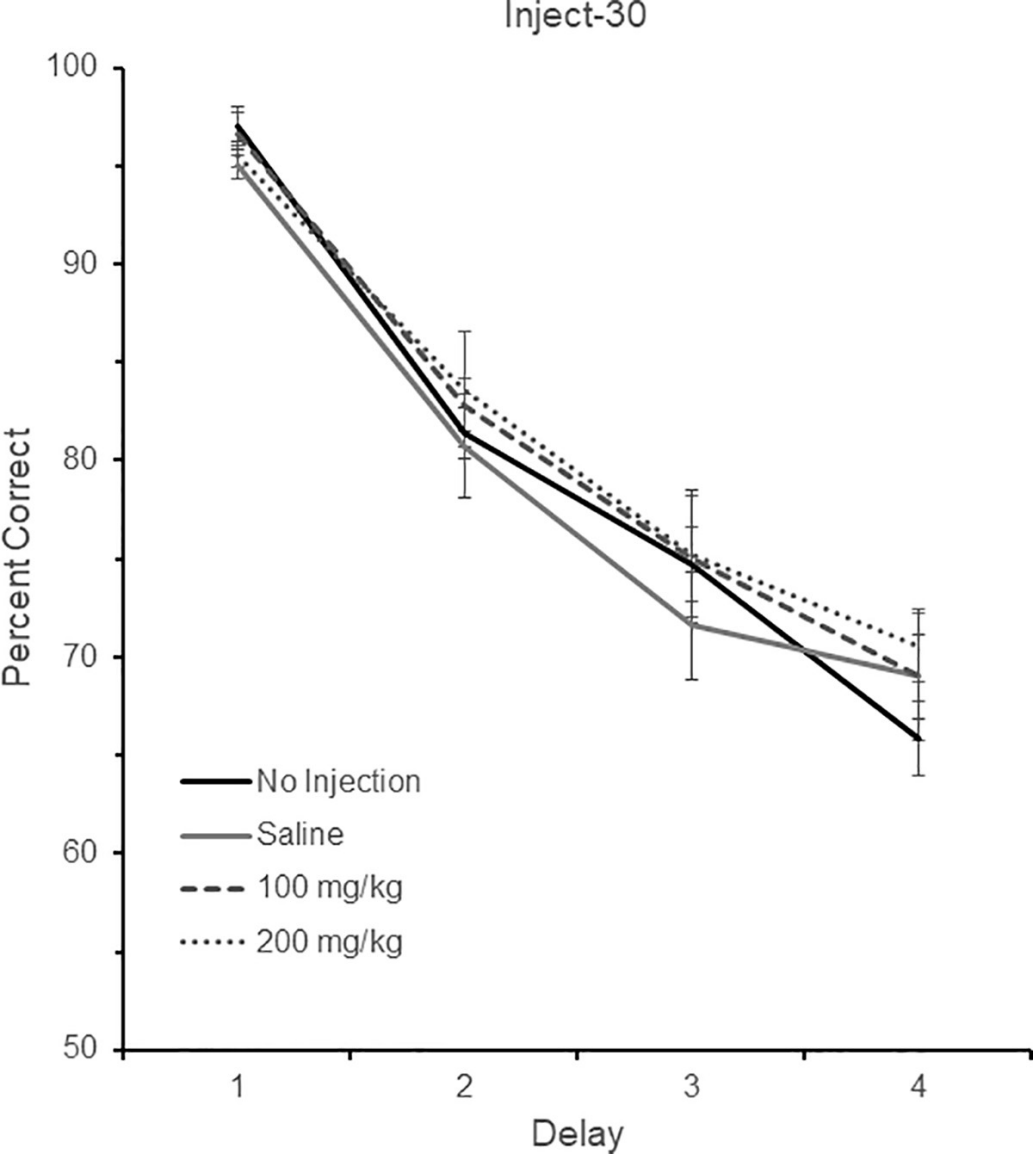
DMS performance in the Inject-30 condition. DMS performance following no injection, saline, 100 mg/kg aniracetam, and 200 mg/kg aniracetam in the Inject-30 condition. A total of six birds were tested. Three birds were tested with delays of 0, 2, 4, and 8 sec (Delay set 2), and three were tested with delays of 0, 3, 6, and 12 sec (Delay set 3). Chance is 50% correct. Error bars are +/- SEM.

Six birds were tested in the Inject-30 group. Three birds were tested with delays of 0, 2, 4, and 8 sec (Delay set 2), and three were tested with delays of 0, 3, 6, and 12 sec (Delay set 3). The performance of these six birds across the four different drug conditions (no injection, saline, 100mg/kg, and 200mg/kg) and their respective four different delay durations is shown in Fig 3. There was a significant effect of delay, *F* (3, 15) = 99.93, *p* < 0.001, indicating that performance fell as the delay increased. There was no effect of either drug, *F* (3, 15) = 1.35, *p* = 0.30, or Drug x Delay interaction, *F* (9, 45) = 0.66, *p* = 0.74.

In addition to performance, we also examined whether aniracetam affected the subjects’ response latency to make a correct choice from the comparison stimuli. There was no effect of delay, *F* (3, 15) = 0.19, *p* = 0.81, drug, *F* (3, 15) = 1.54, *p* = 0.27, or Drug x Delay interaction, *F* (9, 45) = 0.99, *p* = 0.38.

### Inject-60

**Fig 4:**
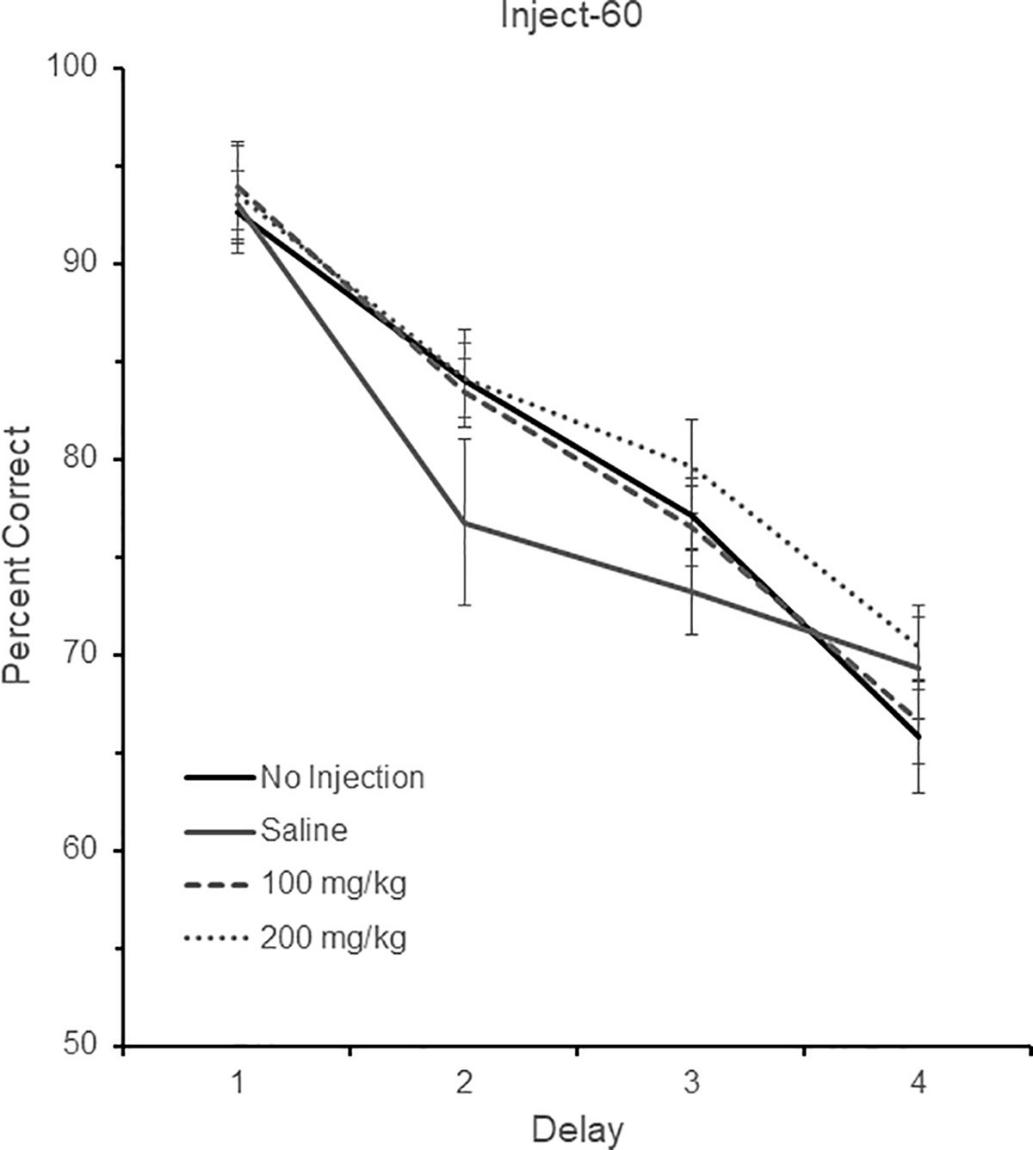
DMS performance in the Inject-60 condition. DMS performance following no injection, saline, 100 mg/kg aniracetam, and 200 mg/kg aniracetam in the Inject-60 condition. A total of eight birds were tested. Four birds were tested with delays of 0, 1, 2, and 4 sec (Delay set 1), one was tested with delays of 0, 2, 4, and 8 sec (Delay set 2), two were tested with delays of 0, 3, 6, and 9 sec (Delay set 3) and one was tested with delays of 0, 5, 10, and 20 sec (Delay set 5). Chance is 50% correct. Error bars are +/- SEM.

Eight birds were tested in the Inject-60 group, all different from those in the Inject-30 group. Four birds were tested with delays of 0, 1, 2, and 4 sec (Delay set 1), one was tested with delays of 0, 2, 4, and 8 sec (Delay set 2), two were tested with delays of 0, 3, 6, and 9 sec (Delay set 3) and one was tested with delays of 0, 5, 10, and 20 sec (Delay set 5). The performance of these eight birds across the four different drug conditions (no injection, saline, 100mg/kg, and 200mg/kg) and their respective four different delay durations is shown in Fig 4. There was a significant effect of delay, *F* (3, 21) = 64.53, *p* < 0.001, indicating again that performance fell as the delay increased in length. There was no effect of either drug, *F* (3, 21) = 2.63, *p* = 0.11, or Drug x Delay interaction, *F* (9, 63) = 1.76, *p* = 0.19.

With respect to the latency data, there was no effect of delay, *F* (3, 15) = 1.41, *p* = 0.28, drug, *F* (3, 15) = 1.55, *p* = 0.25, or Drug x Delay interaction, *F* (9, 63) = 0.58, *p* = 0.51.

### Oral-30

**Fig 5:**
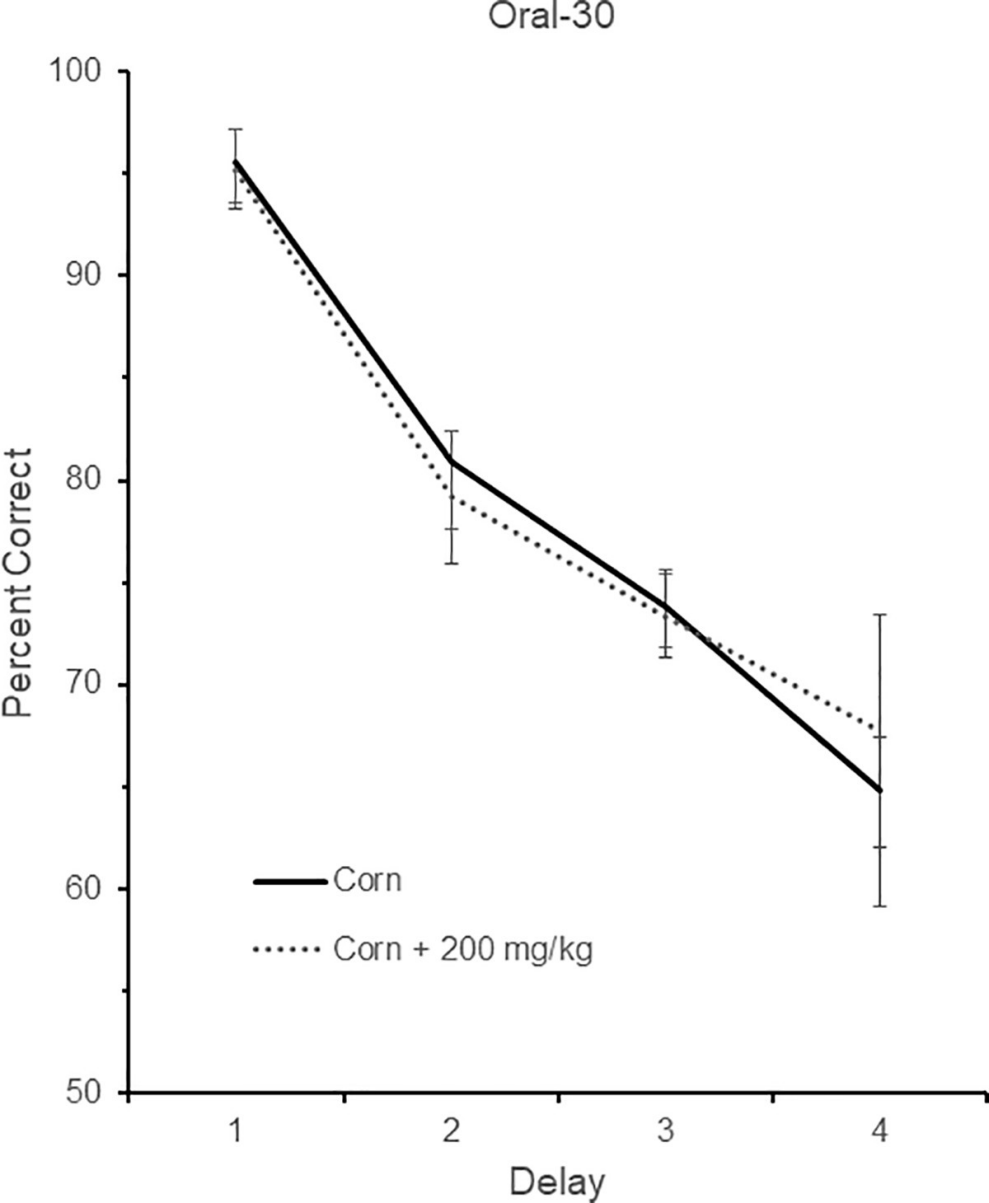
DMS performance in the Oral-30 condition. DMS performance following no drug (corn) or drug (corn + 200 mg/kg aniracetam in the Oral-30 condition. A total of five birds were tested. One bird was tested on delays of 0, 1, 2, and 4 sec (Delay set 1), one was tested on delays of 0, 2, 4, and 8 sec (Delay set 2), two were tested on delays of 0, 3, 6, and 9 sec (Delay set 3), and one was tested on delays of 0, 5, 10, and 20 sec (Delays set 5). Chance is 50% correct. Error bars are +/- SEM.

Five birds were tested in the Oral-30 group. Three of the birds were drawn from birds in the Inject-30 group, and two were drawn from birds in the Inject-60 group. One bird was tested on delays of 0, 1, 2, and 4 sec (Delay set 1), one was tested on delays of 0, 2, 4, and 8 sec (Delay set 2), two were tested on delays of 0, 3, 6, and 9 sec (Delay set 3), and one was tested on delays of 0, 5, 10, and 20 sec (Delays set 5). The performance of these five birds across the two different drug conditions and their respective four different delay durations is shown in Fig 5. There was again a significant effect of delay, *F* (3, 12) = 61.27, *p* < 0.001, indicating that performance fell as the delay increased in length. There was no effect of either drug, *F* (1, 4) = 0.003, *p* = 0.96, or Drug x Delay interaction, *F* (3, 12) = 0.68, *p* = 0.58.

With respect to the latency data, there was no effect of delay, *F* (1, 4) = 0.00, *p* = 0.99, drug, *F* (1, 4) = 0.00, *p* = 0.98, or Drug x Delay interaction, *F* (3, 12) = 0.80, *p* = 0.47.

### Oral-60

**Fig 6:**
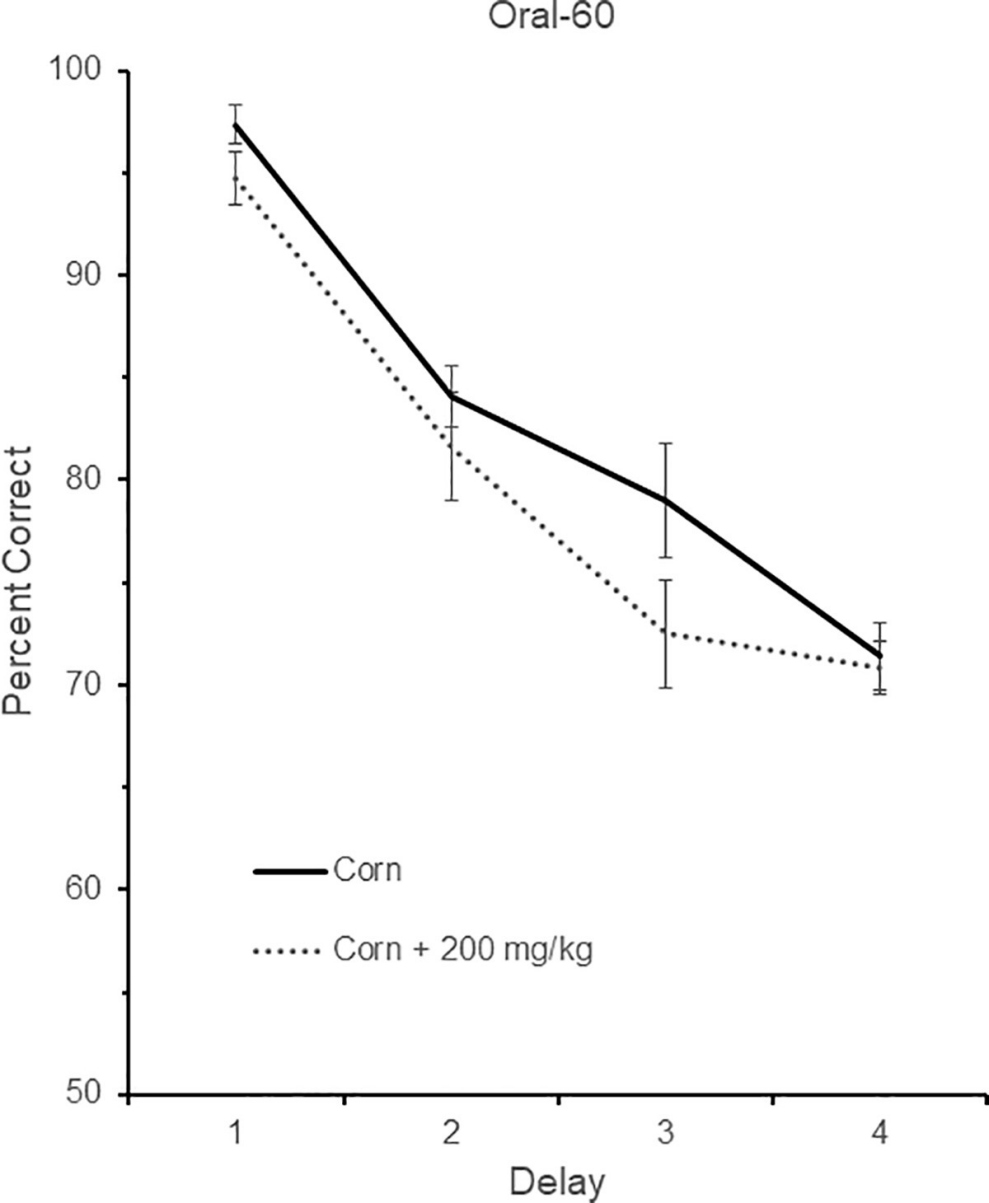
DMS performance in the Oral-60 condition. DMS performance following no drug (corn) or drug (corn + 200 mg/kg aniracetam in the Oral-60 condition. A total of five birds were tested. One bird was tested on delays of 0, 1, 2, and 4 sec (Delay set 1), two were tested on delays of 0, 2, 4, and 8 sec (Delay set 2), and two were tested with delays of 0, 3, 6, and 12 sec (Delay set 3). Chance is 50% correct. Error bars are +/- SEM.

Five birds were tested in the Oral-60 group. Three of the birds were drawn from birds in the Inject-30 group, and two were drawn from birds in the Inject-60 group. One bird was tested on delays of 0, 1, 2, and 4 sec (Delay set 1), two were tested on delays of 0, 2, 4, and 8 sec (Delay set 2), and two were tested with delays of 0, 3, 6, and 12 sec (Delay set 3). The performance of these five birds across the two different drug conditions and their respective four different delay durations is shown in Fig 6. There was again a significant effect of delay, *F* (3, 12) = 84.53, *p* < 0.001, indicating that performance fell as the delay increased in length. There was a significant effect of drug in that aniracetam reduced performance, *F* (1, 4) = 12.67, *p* < 0.05, but no Drug x Delay interaction, *F* (3, 12) = 2.28, *p* = 0.17.

As in all the other latency conditions, there was no effect of delay, *F* (3, 12) = 2.86, *p* = 0.15, drug, *F* (1, 4) = 0.00, *p* = 0.98, or Drug x Delay interaction, *F* (3, 12) = 1.87, *p* = 0.23.

### Pharmacokinetic study

**Fig 7:**
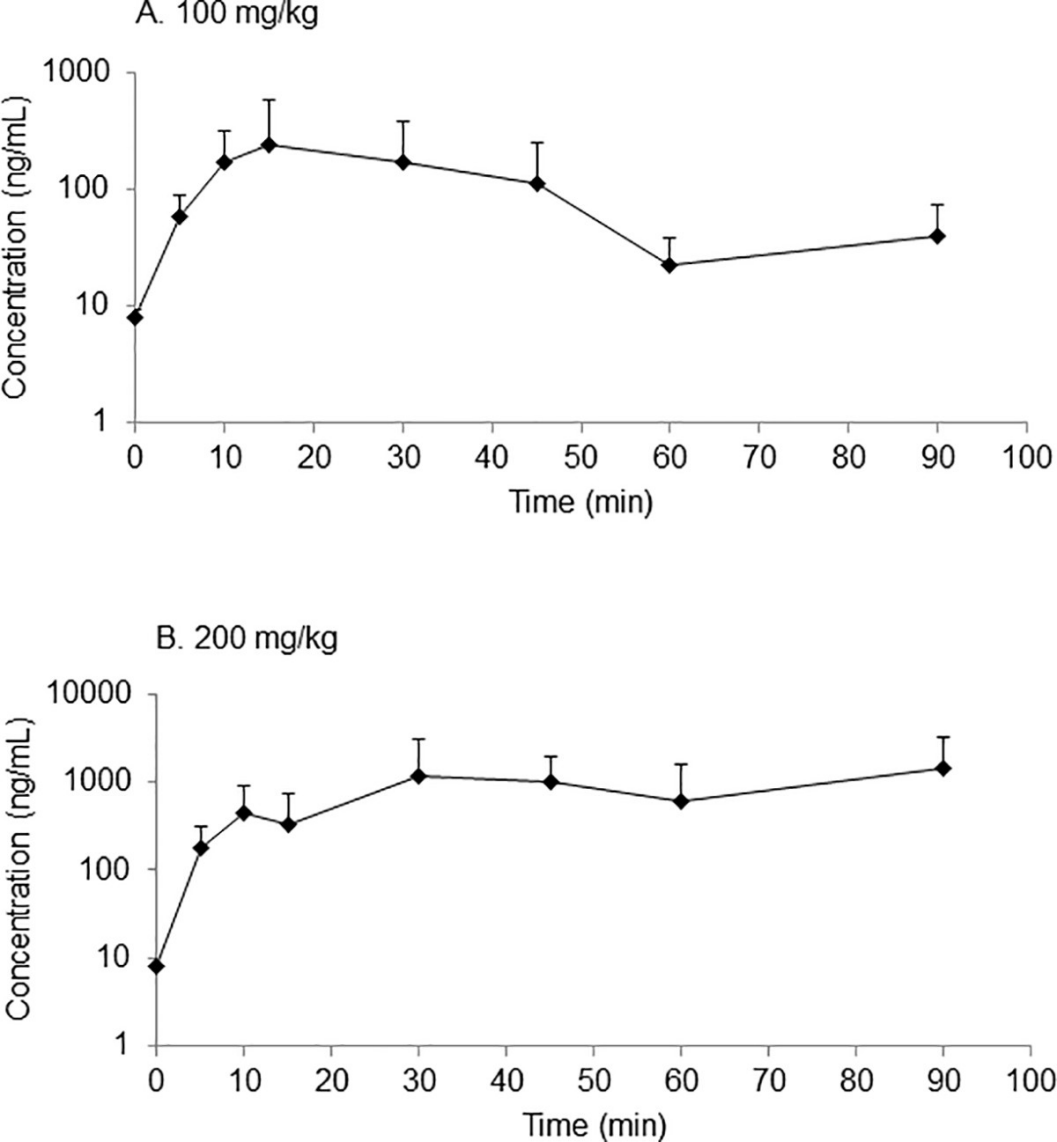
Plasma concentration time curves for aniracetam. Plasma concentration time curves for aniracetam after oral administration of (a) 100 mg/kg aniracetam and (b) 200 mg/kg aniracetam to pigeons. Data points are mean + SD (*n* = 2–5).

The plasma concentration-time curves for aniracetam after oral administration of 100 mg/kg and 200 mg/kg are presented in Fig 7. Following administration of 100 mg/kg aniracetam, the plasma profile was biphasic with an absorption phase until the peak plasma concentration of 243 ng/mL was reached at 15 min after dosing (Fig 7A). Absorption of aniracetam was incomplete over the 90 min period of sampling (Fig 7B) consistent with the calculated MRT of > 30 h (Table 2). For the 200 mg/kg aniracetam dose, plasma concentrations were higher, but not linearly so, than the 100 mg/kg dose with AUC_(0–90)_ of 74,927 ng·h/mL and 8887 ng·h/mL for the 200 and 100 mg/kg dose, respectively (Table 2).

**Table 2:** Pharmacokinetic parameters of aniracetam and *N*-anisoyl-GABA following oral administration of aniracetam to pigeons.

Pharmacokinetic parameter	Aniracetam	*N*-anisoyl-GABA
100 mg/kg		
AUC_last_ (ng·h/mL)	9,309.62	173,740
AUMC (ng·h^2^/mL)	274,098.78	8,633,225
C_max_ (ng/mL)	242.96	4,254.87
T_max_ (min)	15	10
MRT (h)	29.44	49.69
200 mg/kg		
AUC_last_ (ng·h/mL)	74,927	311,055
AUMC (ng·h^2^/mL)	4,082,985	1.9 x 10^7^
C_max_ (ng/mL)	1,463.60	6379.44
T_max_ (min)	90	90
MRT (h)	54.49	60.88

**Fig 8:**
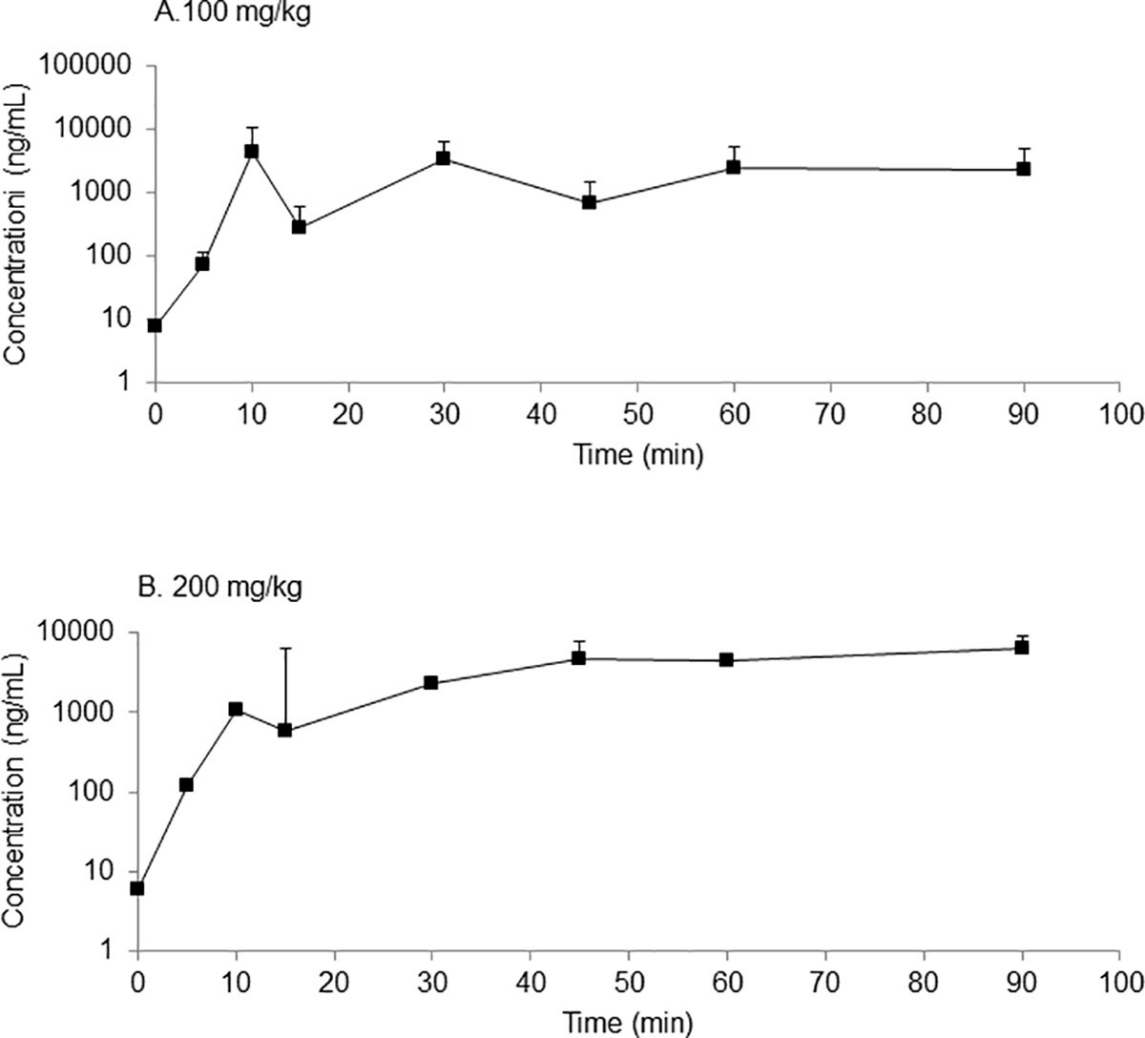
Plasma concentration time curves for *N*-anisoyl-GABA. Plasma concentration time curves for *N*-anisoyl-GABA, the main metabolite of aniracetam, after oral administration of (a) 100 mg/kg and (b) 200 mg/kg aniracetam to pigeons. Data points are mean + SD (*n* = 2–5).

One of the main active metabolites of aniracetam, N-anisoyl-GABA, was detected in the plasma of pigeons approximately 10 min after oral administration of aniracetam (Fig 8). For both doses of aniracetam, there was a biphasic profile, the concentration of N-anisoyl-GABA increasing to a plateau by about 30–40 minutes.

## Discussion

There was no indication that aniracetam improved short-term memory in pigeons, evidenced by a lack of increase in matching accuracy at any of the delays used on the DMS task. Additionally, the absence of any improvement was observed for both the intramuscular and oral delivery routes, and similarly there was no improvement when aniracetam was administered at either 30 or 60 minutes before testing. The lack of improvement was not due to a floor or ceiling performance effect, as the retention functions displayed on the DMS task were ideal for observing any improvement. Finally, the results of the pharmacokinetic study suggest that, at least for the oral administration, we chose the correct testing times in relation to the time-points at which the blood aniracetam and N-anisoyl-GABA concentrations were highest.

In humans, rats, and dogs, aniracetam is rapidly and completely absorbed from the gastrointestinal tract following oral administration and is extensively metabolised in the liver, primarily to *N*-anisoyl-GABA [27, 28]. There is however, a wide variability reported in the literature for the maximum plasma concentrations following oral administration of aniracetam [25, 29]. The present study is the first to investigate the pharmacokinetics following oral administration aniracetam in pigeons and the plasma concentrations observed were within the range reported [29, 30], however were higher than that reported for rats [25]. The concentrations of *N*-anisoyl-GABA were higher in the present study than reported for rats [25] and humans [26]. Investigations into the pharmacokinetics following IV administration is needed for pigeons, however there are currently methodological limitations due to the low solubility of aniracetam. Consequently, subsequent studies are needed to produce a formulation that can be used for IV administration in pigeons.

Mean retention times observed here were considerably longer than the 1–1.5 h reported for rats [25], and the AUCs were greater. It is possible that slow absorption in the pigeon, and differences in metabolism between pigeons and rats, may account for these differences. Gastrointestinal anatomy, physiology and transit time will influence the availability of orally administered drugs and these features vary between species [31]. A characteristic feature of the gastrointestinal tract in avian species not found in mammals is the presence of a crop. The crop is located prior to the proventriculus (stomach) and this can serve to retain orally administered dosage forms and so delay absorption. The birds were fasted in this study, which should have facilitated the direct transit of orally administered material to the proventriculus, however this may not have been complete. Further, the form in which aniracetam is administered (suspension or solid) will impact the rate of absorption, with a suspension resulting in faster absorption.

With respect to the effects of aniracetam on DMS performance, there are several possible reasons why we failed to observe an effect. One is that, due to the dose-dependent nature of previous enhancements noted[11, 13, 16, 24], the doses we chose (100 and 200 mg/kg) were outside the optimal range to observe an effect in pigeons. We selected these doses to cover a higher range than had been previously tested in pigeons (12.5, 25 and 50 mg/kg), which had not elicited any improvement in performance on the DMS task [16]. Indeed, if anything, in the current study 200 mg/kg delivered orally after 60 minutes (but not 30 minutes) had a detrimental effect on performance, possibly because of some of the side effects mentioned earlier (anxiety, insomnia, diarrhoea), and possibly because these side effects would have been more pronounced at 60 minutes post ingestion than 30 minutes post ingestion. It is therefore possible that the optimal dose for enhancement lies somewhere between the doses used in the previous study and those used in the current study. Future research should investigate doses in the 50–100 mg/kg range and possibly also smaller increments to observe where, if any, enhancement occurs. One must also consider the possibility that aniracetam may simply have different effects in an avian brain compared to a mammalian brain. That said, the evidence that aniracetam may lead to improvements in neurologically healthy mammalian brains is weak at best. Finally, there is always the possibility that a larger sample size would reveal and effect, but we suspect if that were true then, given our findings, that effect would be small at best.

Another difficulty in drug treatment is determining the best time to administer the drug in relation to testing times. Aniracetam has a short half-life [32] and, in a previous study no effect was found when pigeons were treated with aniracetam administered intramuscularly 50 minutes before testing[16]. Given that it is possible that the aniracetam had already been metabolised by the time testing occurred, we tested both 30-minute and 60-minutes time-points, and conducted a pharmacokinetic study to determine whether our testing times were appropriate based on the peak blood concentration levels of aniracetam. While the results of the pharmacokinetic study did indicate that peak concentration levels were occurring just before testing began, it did not guarantee that aniracetam was getting into the brain in a timely manner, so future research should investigate a wider array of times for administering the drug. Amakusa et al. [33] report that the metabolite *N*-anisoyl-GABA was found in the cerebrospinal fluid of elderly patients when administered 200 mg aniracetam, providing evidence that aniracetam and/or its metabolites are able to traverse the blood-brain barrier. Further studies could be conducted in pigeons to investigate the availability of aniracetam and *N*-anisoyl-GABA in the brain, perhaps by collecting brain tissue at different time-points and correlating this to the disappearance of these compounds in the blood.

There is growing evidence supporting the restorative effect of aniracetam in animal models of disease or injury. Aniracetam has been shown to reverse impairment caused by scopolamine during a passive avoidance task [15], help to restore lost memory function following trauma to the brain [34], and improve memory function in aged individuals [18]. Similarly, aniracetam has a protective effect when administered prior to scopolamine or CO_2_ disruption [11]. The restorative and protective effects were dose-dependent, with the optimal dose being around 50 mg/kg. The evidence seems convincing, therefore, that aniracetam does improve performance in brains that have been subject to insult.

Whether aniracetam can improve memory performance in healthy brains is much less certain. Neurologically-healthy monkeys treated with aniracetam demonstrated improved performance on a DMS task [16, 23], and rats experienced an increased ability to discriminate between novel and familiar objects following aniracetam (50 mg/kg) administration [18]. Rats also exhibited improved performance on the radial arm maze test when treated with high doses (400 and 800 mg/kg) of aniracetam [24], and improved performance on an active avoidance task when given aniracetam (50 mg/kg) 60 minutes before testing [13]. That all said, the improvements, although significant, are often small, and are observed in a varying range of doses, making it difficult to determine where exactly the enhancement, if any, occurs. For example, a study using rats failed to find an enhancement at doses lower than 400 mg/kg, but did notice an enhancement at 400 and 800 mg/kg [24], whereas another study in rats found an enhancement at 50 mg/kg, but not at 25 or 100 mg/kg [18].

A few recent studies have found that aniracetam conveys no therapeutic benefit in healthy mice with respect to a variety of learning and memory paradigms[35, 36]. After treatment with aniracetam, mice learned to balance on an accelerating rotating rod at the same rate as mice treated with a placebo [35]. Aniracetam treatment did not improve spatial learning and memory in the Morris water maze, and did not enhance fear conditioning[35, 36]. Our current study, as well as an earlier study in pigeons [16] are in line with these findings, and support the view that aniracetam does not improve learning and memory in healthy subjects. Aniracetam appears to be well suited towards improving memory in an impaired model of memory, and this is reflected in its current use as treatment for dementia. If the goal, however, is for enhanced memory above healthy levels, we may need to look to other AMPAkines. Indeed, there are a number of other AMPAkines that are showing promising results in improving memory in healthy subjects. CX516 (1-(quinoxaline-6-ylcarbonyl)piperidine) improved performance on a delayed non-matching-to-sample task, and was observed to have longer-lasting effects, with performance remaining high for seven days following cessation of treatment[37]. In humans, those treated with CX516 had a heightened ability to recall nonsense syllables, whereas those treated with a placebo experienced a decay in recall[38]. Similarly, the AMPAkines CX717 and Org 26576 have the potential to be effective cognitive enhancers, as they have both improved performance on memory tests in neurologically-healthy animals[39, 40].

## Conclusion

Cognitive enhancers are a new and exciting area of research that is rapidly developing. One family of drugs, AMPAkines, show the most potential for cognitive enhancement, via modulation of the AMPA receptor, and with minimal negative side effects. The current study failed to find an improvement in matching accuracy in healthy pigeons after treatment with one such AMPAkine, aniracetam. Matching accuracy did not improve at doses of 100 mg/kg or 200 mg/kg. We tested both oral and intramuscular routes of administration, and tested two different time-points of drug delivery; 30 minutes and 60 minutes prior to testing. We also conducted a pharmacokinetic study to determine the time after oral administration at which blood concentration was highest and found that our chosen testing times were in line with these times. Despite the negative results of the present study, they add to the argument being formed by the current literature that, while effective at restoring memory after experimentally-induced disruption, aniracetam has little effect on memory performance in healthy subjects. What little evidence there is for an improvement in healthy subjects is marginal at best, and is inconsistent in terms of when and how much of the drug should be administered to see an effect.

## Supporting information

S1 File Supporting information. (XLSX)
